# *Steps that count!* : The development of a pedometer-based health promotion intervention in an employed, health insured South African population

**DOI:** 10.1186/1471-2458-12-880

**Published:** 2012-10-17

**Authors:** Julian D Pillay, Tracy L Kolbe-Alexander, Karin I Proper, Willem van Mechelen, Estelle V Lambert

**Affiliations:** 1 UCT/MRC Research Unit for Exercise Science and Sports Medicine, Faculty of Health Sciences, University of Cape Town, Cape Town, South Africa; 2 Department of Basic Medical Sciences, Faculty of Health Sciences, Durban University of Technology, Durban, South Africa; 3 Department of Public and Occupational Health, EMGO Institute for Health and Care Research, VU University Medical Centre, Amsterdam, Netherlands

**Keywords:** Pedometer, Health risk appraisal, Physical activity, Computer-based feedback

## Abstract

**Background:**

Physical activity (PA) has been identified as a central component in the promotion of health. PA programs can provide a low cost intervention opportunity, encouraging PA behavioral change while worksites have been shown to be an appropriate setting for implementing such health promotion programs. Along with these trends, there has been an emergence of the use of pedometers as a self-monitoring and motivational aid for PA.

This study determines the effectiveness of a worksite health promotion program comprising of a 10-week, pedometer-based intervention (“*Steps that Count!*”), and individualized email-based feedback to effect PA behavioral change.

**Methods:**

The study is a randomized controlled trial in a worksite setting, using pedometers and individualized email-based feedback to increase steps per day (steps/d). Participant selection will be based on attendance at a corporate wellness event and information obtained, following the completion of a Health Risk Appraisal (HRA), in keeping with inclusion criteria for the study. All participants will, at week 1 (pre-intervention), be provided with a blinded pedometer to assess baseline levels of PA. Participants will be provided with feedback on pedometer data and identify strategies to improve daily PA towards current PA recommendations. Participants will thereafter be randomly assigned to the intervention group (INT) or control group (CTL). The INT will subsequently wear an un-blinded pedometer for 10 consecutive weeks.

Individualized feedback messages based on average steps per day, derived from pedometer data (INT) and general supportive/motivational messages (INT+CTL), will be provided via bi-weekly e-mails; blinded pedometer-wear will be conducted at week 12 (post-intervention: INT+CTL).

**Discussion:**

The purpose of this paper is to outline the rationale behind, and the development of, an intervention aimed at improving ambulatory PA through pedometer use, combined with regular, individualized, email-based feedback. Pedometer-measured PA and individualized feedback may be a practical and easily applied intervention.

**Trial registration:**

Number: DOH-27-0112-3951

## Background

Physical inactivity is a global health concern. Despite the awareness of physical inactivity, solutions to the problem are complex, as behavioral change is often difficult to achieve and, more importantly, to sustain. Small behavioral changes may, however, be more feasible to achieve and to maintain, the impact of which may be significantly beneficial towards improved clinical outcomes and overall well-being [1]. The work-site is an appropriate setting to initiate health promotion (HP) intervention programs where social and cultural disparities can often be overcome through the shared interest of well-being [2]. A critical review [3] identifying the effectiveness of work-site physical activity programs on physical activity, physical fitness, and health showed support towards the implementation of worksite physical activity programs to increase the habitual levels of physical activity (PA) among employees.

In South Africa (SA), a large proportion of the working population can be reached through health risk appraisals (HRA’s), conducted by health insurers [4]. This voluntary appraisal typically consists of anthropometric measures (such as body mass index (BMI) and waist circumference); clinical measures (such as blood glucose and blood cholesterol concentrations, blood pressure (BP)) and information related to health risk behavior, including physical activity (PA) and readiness for change. Similar HRA’s are administered in other countries such as the Netherlands [5] and Denmark [6] and more recent studies have adopted this approach as part of an intervention to evaluate the effectiveness of HRA’s and follow-up support [7,8].

The emergence of pedometers as a useful self-monitoring and feedback tool and therefore a useful motivational aid for increasing PA [9,10] has complemented behavioral change strategies with the objective of increasing PA. Researchers have acknowledged that in terms of practicality, pedometers offer a good solution for a low cost, objective monitoring and behavioral modification tool and a practical aid for PA interventions [11-14]. Pedometers have therefore gained popularity for use in PA interventions in various settings [15] to facilitate behavioral change.

Providing individualized feedback has been promoted as a useful adjunct to many health and well-being interventions and has often been used as an additional support measure to pedometer-based interventions [16]. A number of on-site and face-to-face programs have been found to be effective [3,17]. There is, however, a large gap between the development of effective interventions and their feasibility for use in public health practice [18,19]. A primary limitation is the high cost and large time demands on both staff and participants [19]. Using lower cost intervention strategies, such as pedometer-based approaches supplemented by email-based feedback, may have the potential to overcome this limitation. Also, an attempt at evaluating the benefits of short-term interventions (such as a 10 week intervention) may be useful in identifying whether significant changes in PA behavior can be achieved within this time-frame.

This study provides an opportunity to evaluate the effect of a pedometer-based intervention complemented by individualized, email-based feedback in improving PA in an employed population.

### Aim

To develop a 10-week, pedometer intervention*- “Steps that count!”-* that examines the effectiveness of pedometer use complemented by individualized, email-based feedback on daily PA levels, in an employed South African population.

## Methods

The proposed study is a randomized controlled study on the effectiveness of “*Steps that count!”* in a worksite setting, primarily using pedometers and feedback messages through regular, bi-weekly emails.

The concept of *“Steps that count!”* is developed from the findings of 2 recent studies that identify and highlight the importance of intensity of steps accumulated [20,21]. This (intensity-based steps) outcome complements other recent pedometer-based studies that have identified and recommended steps/min rates for moderate physical activity (MPA) [14,22-24]. A pilot feasibility study recently conducted [25], using a similar methodology, has further informed the development of this intervention and sample size calculation. The term “S*teps that count!”,* and the intervention presented has therefore been adopted as a term and a strategy for engaging people into accumulating intensity-based steps, and forms a key element of the intervention.

The behavioral strategies underlying “*Steps that count!”* include certain principles from several behavioral theories such as the theory of planned behavior and reasoned action [26,27], which proposes that a person’s intention to perform a behavior is the central determinant of performing that behavior. In addition, the Transtheoretical Model (TTM) [28], developed as an explanatory framework for intentional behavioral change, is based on the observation that people tend to move through a series of stages (pre-contemplation, contemplation, preparation, action and maintenance) in their attempt to change a certain behavior [29]. This intervention is specifically targeted at individuals in the contemplation phase of the TTM, i.e. individuals considering change even though they may be ambivalent about changing.

**Figure 1:**
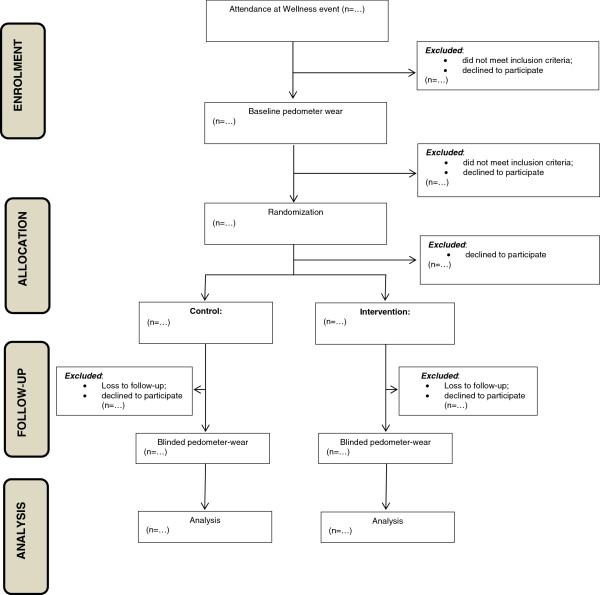
Flow of the study design.

The intervention is not designed to test any particular theoretical model, but rather to incorporate elements from these models to initiate and sustain behavioral change. These behavioral strategies will be applied in a basic structure to improve PA by providing cues and repetition that help make the new behaviors habitual (Figure 1). Following the baseline pedometer wear, “*Steps that count!”* promotes and reinforces the intention to change PA behavior, through feedback on PA from the pedometer data, a brief discussion around current PA recommendations, and identifying possible strategies to improve steps/day. The intervention attempts to motivate an increase in PA by requesting commitment to small achievable goals, such as “adding 10 minutes of ‘*steps that count’* to your day” or “increasing daily steps by 10% per week until 30 minutes of *‘steps that count’* are achieved”. Individual PA patterns are summarized and presented, PA recommendations reinforced and some options as to how to increase PA levels are provided in the emailed feedback.

### Study setting and study population

The study will be conducted at selected worksite settings that are based in the province of KwaZulu-Natal, RSA. The organization will offer and conduct a HRA to employees attending the wellness event. The HRA will identify cardiovascular risk factors (including: family history, dietary intake behavior, smoking and stress), and biometric measures will include BMI, %BF, BP, blood glucose and blood cholesterol concentrations. In addition, self-reported PA as well as intention for change towards improved PA will be assessed. After completion of the HRA, employees will be invited by the researcher and/or assistant to participate in the study (subject to meeting the relevant inclusion criteria). All employees eligible to participate in the study will be requested to wear a blinded pedometer for 7 consecutive days as a baseline measure of PA.

### Sample size, recruitment and randomization

We estimated sample size on the basis of aiming to show an improvement of 2,500 steps/d (and a baseline value of 7,500 steps/d) with an approximate standard deviation of ±3,000 steps/d (as established through recently published papers and systematic reviews of pedometer-based interventions conducted from 1966–2007 [30-32], as well as using the outcomes from recent pedometer studies conducted in RSA [20,25]). A sample size of 30 participants per arm of the study is required to ensure 80% statistical power and with a p-value set at <0.05. However, if a modest improvement of 1 500 steps/d is considered, a sample size of approximately 85 participants per arm is required. Considering this possibility and the likelihood of performing sub-group analyses of the data based on factors such as age and gender, a sample size of 150 participants in the INT and CTL respectively would be an appropriate estimate to account for these factors.

In order to achieve this, 1200 employees attending wellness events will be targeted. Of these, a minimum of 480 employees (40%) will be identified to be in the contemplation stage of TTM [33]. On an assumption that at least eighty percent (N=385) will agree to participate (as in the contemplation stage of TTM for PA), of which approximately 90% (N=345) will complete a 3 day blinded pedometer wear [34], 175 participants will be randomly assigned to the INT and wait-listed CTL respectively. An expected 15-20% loss to follow-up [5,35] will result in a final number of approximately 150 per arm of the study for analyses.

### Inclusion and exclusion criteria

Employees attending the wellness event and willing to be included in the study will be eligible to participate. Other inclusion criteria include: being between the age of 21 years (inclusive) and 50 years (exclusive); being identified as in the contemplation stage of TTM towards improved PA and having a contract with employer until end of the 12-week measurement period so that completion of the intervention is possible. Employees will be excluded for the following reasons: pregnancy; diagnosis or treatment of cancer; any other condition that makes PA difficult/impossible; contract workers whose employ with the company will end before the follow up measurement at week 12; non-compliance of a minimum 3 day (including 1 weekend day) blinded pedometer wear.

### Ethical considerations and pre-participation screening

This study will be conducted in accordance with the Declaration of Helsinki, Good Clinical Practice as well as the ethical laws of SA. Ethical approval for the study was obtained from the Human Research Ethics Committee of the Faculty of Health Sciences, University of Cape Town (UCT), SA (reference number: 044/2009) and the study has been registered by the SA Department of Health (DOH-27-0112-3951). Following agreement to participate in the study, the Physical Activity Readiness Questionnaire (PAR-Q) [36] will be administered to ensure that there are no health risks to improving PA levels during and after the intervention. Employees who agree to participate in the study will be asked to sign an informed consent form prior to participating in the study. Participants will be assured that their participation in the study is voluntary and that they may withdraw at any time. They will also be reassured that their withdrawal will not have any negative impact on their employment, and that they will continue to receive all usual care health insurance benefits and/or programs. The participants will also be assured that their employer will not have access to any of the information collected for the research study, and that all information is strictly confidential.

### Testing protocol

All eligible employees who sign the informed consent will be required to wear a blinded pedometer (Omron HJ 720 ITC) for 7 days during week 1 and week 12 of the study. Upon return of the pedometer (after week 1), steps/d data will be electronically downloaded by the researcher according to the Omron Health Management Manager software protocol [37] and feedback (in terms of average total steps/d and information relating to moderate intensity steps (*“steps that count”*)) will be provided to each participant. Simple messages to improve PA levels will be discussed in keeping with the PA recommendation of 30 minutes of moderate PA (MPA) at least five times a week [38]. Participants will be encouraged to improve their PA levels steadily (for example by 10% per week until 30 minutes of MPA is achieved) during the subsequent 10 weeks. They will then be randomly allocated to an INT or wait-listed CTL and participants in the INT will be provided with an un-blinded pedometer for the subsequent 10 weeks. Those included in the INT will be guided as to how to download their pedometer data and its interpretation. (A 1-page step by step guideline will be provided and participants will be advised to contact the researcher for assistance, if need be). Participants will be advised to download data whenever suitable (This would provide the researcher with information as to how often the downloading feature was accessed).

Following the blinded pedometer wear at week 12 (INT and CTL), an HRA similar to the initial HRA will be conducted. The results obtained (HRA information and pedometer data) will then be compared with the information obtained in week 1 to establish the intervention effect. Participants in the CTL will be offered the pedometer intervention after the HRA conducted at 12 weeks.

Figure 1 provides a flow diagram of the randomized controlled trial intervention plan.

### Health Risk Appraisal (HRA)

Aspects of the HRA relevant to our study include demographic factors (i.e. age and gender), self-reported volume and intensity of PA, as well as information relating to intention and readiness for change toward improving PA. Additionally, BP, body height and body weight, %BF, BMI and waist circumference will be measured. The HRA will be conducted by qualified, trained staff and will form part of the wellness event conducted.

### Pedometer wear and data recording

Participants will be asked to wear the Omron HJ 750 ITC pedometer, attached to the left or right hip as conventionally worn in most studies [39], by both INT and CTL in weeks 1 and 12 respectively. After baseline measurement (week 1), only the INT will continue with subsequent un-blinded pedometer wear. Following the blinded pedometer wear, the pedometer data will be downloaded electronically by the researcher according to the Omron Health Management Manager Software protocol [37]. One of the unique features of the pedometer is the ability to provide an hourly representation of steps/d. Furthermore, in addition to indicating total steps/d, the output illustrates steps accumulated as being “aerobic” or “non-aerobic” according to the Omron classification that integrates both intensity and duration. A record of steps classified as “aerobic” (≥60steps/min, minimum duration of 1-minute) and “non-aerobic” (<60steps/min and/or <1-minute duration) is therefore provided. Similarly, total time spent accumulating “aerobic” steps in minutes/day (aerobic time) and the number (in hours) of sedentary time can be identified.

The validity and reliability of this brand and model of pedometers has been studied at various mounting positions under prescribed and self-paced walking conditions with both healthy and overweight adults and is suggested as an accurate measure of step counts [40,41].

### Intervention content

Pedometer data will be requested (via e-mail) from the INT at bi-weekly intervals. INT participants will be provided with individualized, emailed feedback and a general pamphlet on ways to increase PA following the bi-weekly receipt of pedometer data, via email. The CTL will similarly be provided with the general pamphlet (as in the INT) at bi-weekly intervals.

**Figure 2:**
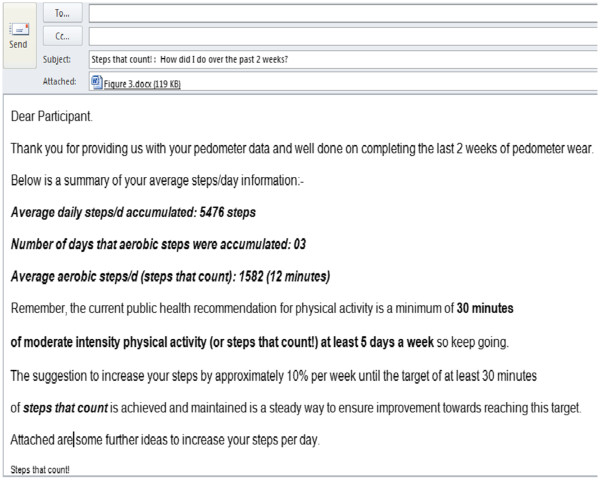
Illustrated feedback characteristics.

The individualized feedback (provided only to the INT) will include information on the average daily steps/d accumulated, the number of days (if any) that aerobic steps were accumulated, and the volume thereof in the form of a personalized email. The feedback will also include information such as the highest number of steps/d accumulated by the individual over the past two weeks and the category within which the average steps/d fall, as per current steps/day categorizations [22,23,42] (Figure 2). Participants will be encouraged to steadily increase their steps by approximately 10% per week until the target of at least 30 minutes of aerobic steps (displayed as “orange” steps on the pedometer download) is achieved and/or maintained by the end of the intervention.

The general supportive/motivational messages will include a key message (such as “be active everyday” or “walk tall”) and a few strategies to achieve this (for example, “Use the stairs instead of the lift/escalator”; “Walk fast enough so as to increase your breathing rate yet not feel out of breath”).

**Figure 3:**
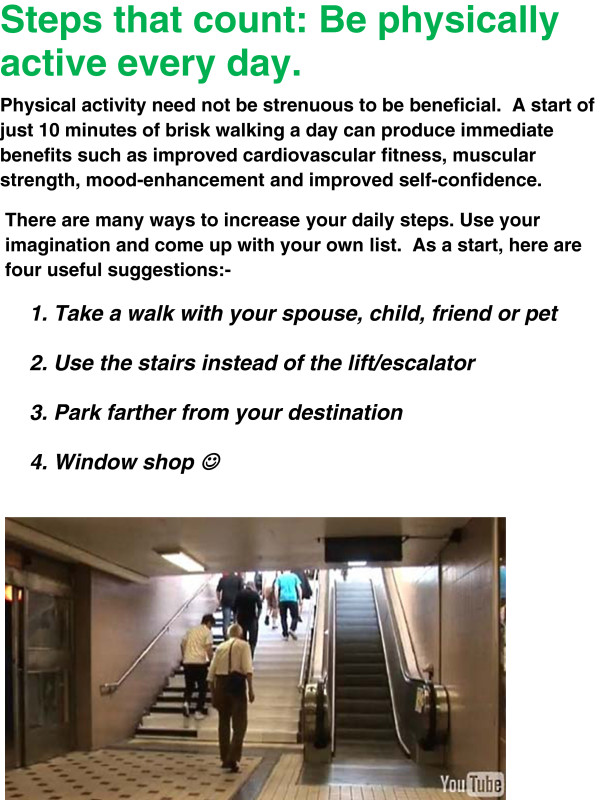
Illustrated general motivational messages.

The purpose of the bi-weekly email is to provide a summation of pedometer-based PA patterns; to remind and reinforce current PA recommendations and to provide some strategies and/or options for “adding steps” to one’s day. Figure 2 and Figure 3 provide examples of a weekly email, respectively.

### Outcome measures

The primary outcome measure (daily PA levels in terms of steps/d) will be assessed at baseline (week 1) and end (week 12) for both the INT and CTL, in order to detect changes in daily PA over time, as a function of the intervention. Data will be derived from the pedometer and expressed as steps/day. More importantly, information on the volume of sustained and moderate-vigorous intensity steps (“*Steps that count”)* will be assessed at baseline (week 1) and at the end of the intervention (week 12) for both the INT and the CTL. Secondary outcomes such as systolic BP and diastolic BP, BMI and %BF (as per clinical measures of HRA) will be assessed at week 1 and week 12 in both groups.

### Statistical analyses

Statistical analyses to determine effectiveness of the intervention will be based on group allocation, regardless of the actual intervention received or of adherence to the intervention, i.e. intention to treat analysis. Linear regression analyses will be performed with the follow-up value of the outcome measure as the dependent variable and adjustment for the baseline value. Assumptions of linear regression analysis will be verified with residual analysis. To assess whether the differences in the primary outcome between the groups are affected by random differences between them, an analysis of covariance (ANCOVA) will also be undertaken. For the process evaluation, descriptive analyses will be conducted among INT only. All analyses will be performed with STATISTICA version 8 (StataSoft Inc., Tulsa, OK, USA) and the significance level will be set at p-values <0.05.

## Discussion

The purpose of this paper is to outline the rationale behind, and the development of, an intervention aimed at improving the daily PA in a South African employed population, and to describe the study protocol evaluating its effect.

A recent literature review identified thirty studies using web-based interventions to increase weight loss and/or PA [43]. Twenty-eight of the studies (93%) reported positive changes in moderate to vigorous PA level, fruit and vegetable intake and psychological factors [43]. The review suggests that web-based interventions are a useful educational tool for increasing awareness and making healthy behavior changes. The self-maintained improvement of PA and maintenance thereof is a further aspect that can also be monitored and evaluated over regular time intervals.

There are limitations that must be noted in the study design. Firstly, there is an element of selection bias as the study will involve selection from a convenience sample of persons who are recruited as a result of attending a corporate HRA. Secondly, the general information provided to both the INT and CTL is the same irrespective of individual progress toward improved PA. Also, the CTL receives the same general motivational messages as the INT, bi-weekly, which may lead to increased PA in the CTL and a resultant weakened effect of the intervention. A further limitation is that the pedometer will be used as a measurement tool (albeit blinded for measurement at weeks 1 and 12, respectively) and during the 10 week intervention.

The study has strengths that can be noted. To our knowledge, this will be the first pedometer-based intervention conducted in SA (other than the pilot feasibility study conducted) and will provide useful information regarding potential for PA improvements through pedometry in an adult working group. This is the first pedometer-based intervention, to our knowledge, that takes into account intensity and duration of PA during free-living wear to provide information on patterns of PA in an employed, adult population. The study will, therefore be useful for further pedometer-based intervention initiatives that can be applied in other contexts and settings on a larger scale.

With a focus on daily PA using individualized, brief feedback and self-monitored, pedometer-measured PA, the success of such an intervention will have widespread public health implications, particularly if shown to produce successful outcomes in the limited extent of external support.

## Competing interests

The authors declare that they have no competing interests.

## Authors’ contribution

All of the authors have made substantive intellectual contributions to the study in terms of the conception and design of the study. All authors have contributed to the write-up of this paper and have read and approved the final version of the manuscript.

## Pre-publication history

The pre-publication history for this paper can be accessed here:

http://www.biomedcentral.com/1471-2458/12/880/prepub
